# An Atypical Presentation of Muscle-Invasive Papillary Urothelial Carcinoma Mimicking Extensive Urethral and Bladder Calculi: A Case Report

**DOI:** 10.7759/cureus.97064

**Published:** 2025-11-17

**Authors:** Ashutosh M Sapre, Lydia Frost

**Affiliations:** 1 Urology, Peterborough City Hospital, Peterborough, GBR

**Keywords:** bladder cancer, case report, intravesical mitomycin c, papillary urothelial carcinoma, turbt, urethral calculi

## Abstract

Bladder cancer commonly presents with haematuria, but its clinical features can vary, and unusual presentations may delay diagnosis. Papillary urothelial carcinoma (PUC) is a frequent subtype, typically arising from the bladder urothelium. While most cases are detected through standard urological symptoms and imaging, the presence of extensive urethral and bladder calculi can obscure the underlying pathology.

We describe a 45-year-old man with a history of recurrent urinary stone passage who presented with haematuria following expulsion of a large urethral calculus. Examination revealed palpable stones along the urethra, and imaging demonstrated multiple sizeable, calcified lesions within the bladder and urethra. Cystoscopy confirmed complete obstruction of the penile and bulbar urethra by stones, which were removed cystoscopically. A large calcified intravesical mass was subsequently excised. Histopathological analysis identified high-grade PUC with invasion of the muscularis propria. The patient received intravesical mitomycin C one day after resection, prior to histology results, which showed muscularis propria involvement. The patient underwent further staging with pelvic MRI, which showed no evidence of advanced or metastatic disease, and a computed tomography urogram to visualise the upper tract, which showed no further stones or masses in the kidneys, ureters or collecting system and complete resection of bladder mass. At follow-up, he remained clinically stable with no signs of recurrence or evidence of metastasis.

This case highlights an uncommon presentation of muscle-invasive PUC concealed by extensive calculi. It emphasises the importance of maintaining diagnostic vigilance in patients with persistent or atypical urolithiasis and demonstrates the value of early histopathological evaluation and timely postoperative intravesical therapy.

## Introduction

Bladder cancer is a significant global malignancy, ranking as the 10th most commonly diagnosed cancer and the 13th leading cause of cancer-related death worldwide [1]. Among its histological subtypes, papillary urothelial carcinoma (PUC) is the most prevalent, comprising approximately 70-80% of urothelial neoplasms [2]. The disease demonstrates a distinct male predominance with a male-to-female ratio of nearly 3:1 and typically affects individuals in their sixth to seventh decades of life [1,2]. Well-established risk factors include tobacco smoking, occupational exposure to aromatic amines, chronic urinary tract inflammation, and prolonged bladder irritation from calculi or indwelling catheters [3,4]. Urolithiasis itself has also been implicated as a potential risk factor for bladder cancer, particularly when chronic or recurrent [4].

Clinically, PUC often presents with painless gross haematuria, but its manifestations can vary widely depending on the tumour location, size, and depth of invasion [2]. Non-muscle-invasive disease generally has a favourable prognosis following complete resection and adjuvant intravesical therapy; however, recurrence is common, and some cases progress to muscle-invasive disease [5].

The coexistence of bladder calculi and urothelial carcinoma is uncommon but clinically important, as chronic irritation and infection may contribute to malignant transformation [3,4]. In rare circumstances, extensive calcification may obscure the underlying malignancy, delaying diagnosis. Urethral and bladder calculi with high-grade PUC appear to be extremely rare; available literature offers limited reports of this malignancy in the setting of large calculi.

We present a rare case of muscle-invasive PUC manifesting as extensive urethral and bladder calculi in a 45-year-old man without traditional risk factors. This case underscores the importance of thorough evaluation of atypical stone disease and timely histopathological confirmation to ensure accurate diagnosis and appropriate management.

## Case presentation

A 45-year-old man with a one-year history of recurrent passage of urinary stones presented with new-onset gross haematuria following the passage of a large urethral calculus. He denied flank pain, fever, nausea, vomiting, or scrotal swelling but reported suprapubic discomfort. Physical examination revealed suprapubic tenderness, and multiple hard nodular structures were palpable along the corpus spongiosum, consistent with urethral calculi. He had no significant past medical history; however, he was a known current smoker with a previous history of alcohol abuse. He had quit 3 to 4 years prior to presentation.

**Figure 1:**
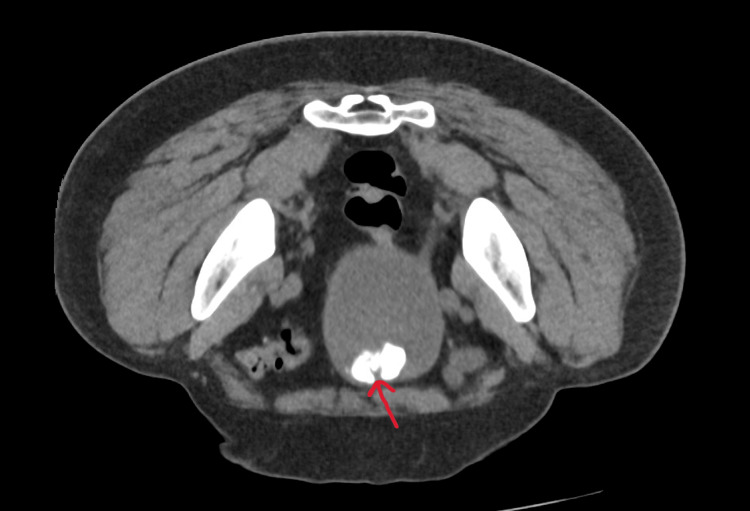
Computed tomography of the kidneys, ureters and bladder showing a large calcification in the bladder (red arrow).

**Figure 2:**
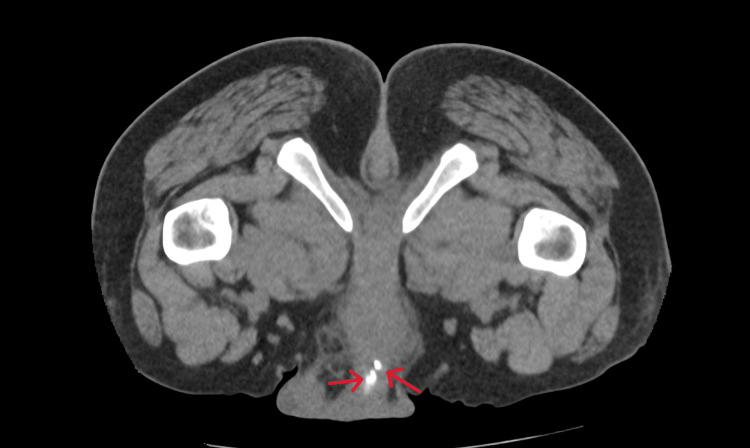
Computed tomography of the kidneys, ureters and bladder showing multifocal calcifications in the urethra suggestive of stones (red arrows).

**Figure 3:**
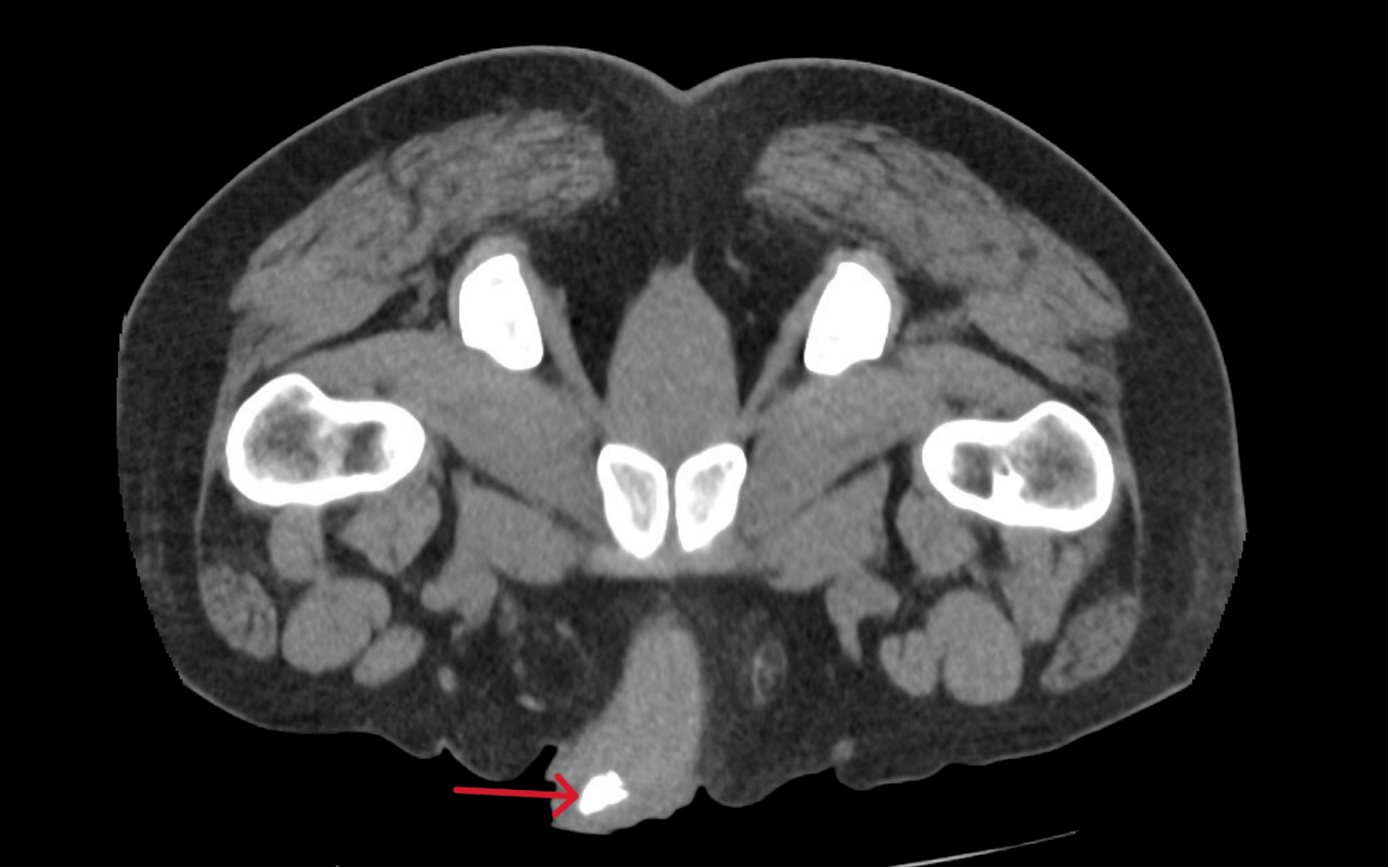
Computed tomography of the kidneys, ureters and bladder (CT KUB) showing a large calcification in the region of the urethra (red arrow).

Routine laboratory testing demonstrated that there was evidence of an inflammatory response with a raised white cell count, neutrophil count and slightly raised C-reactive protein (CRP) count. However, the kidney functions, uric acid and glucose were normal (Table 1). Computed tomography of the kidneys, ureters, and bladder (CT KUB) revealed multiple large heterogeneous calcified structures within the bladder, the largest lesion measuring 50 mm near the left vesico-ureteric junction (Figure 1). A second lesion, located anteriorly, measured 44 mm, and a third inferior lesion measured 12 mm. Multifocal coarse calcifications were also identified along the urethra (Figures 2, 3). No evidence of hydroureteronephrosis or upper tract obstruction was observed.

**Table 1:** Laboratory results from blood tests taken on the day of admission showing raised white cell, neutrophil count and C-reactive protein. Kidney functions, uric acid and glucose are normal. eGFR: estimated glomerular filtration rate

Blood test	Result	Normal range (unit)
C-reactive protein	23	<5 (mg/ml)
White blood cell	21.8	4.0-11.0 (10^9/L)
Haemoglobin	105	115-160 (g/L)
Platelet	694	150-450 (10^9/L)
Neutrophil	17.6	2.0-7.5 (10^9/L)
Calcium	2.38	2.12-2.62 (mmol/L)
Phosphate	0.8	0.8-1.5 (mmol/L)
Sodium	143	135-145 (mmol/L)
Potassium	4.2	3.5-5.3 (mmol/L)
Chloride	111	95-110 (mmol/L)
Creatinine	91	62-115 (μmol/L)
Urea	5	2.5-7.8 (mmol/L)
eGFR	87	60-90 / >90 (ml/min/1.73m^2^)
Uric Acid	270	200-430 (μmol/L)
Glucose	5.5	4-7 (mmol/L)

Urine dip of the patient showed darker colour with haematuria, slightly acidic pH and other markers consistent with stone damage of urothelium but no infection (Table 2).

**Table 2:** Urine dip findings. Haematuria commonly presents with stones. Some protein is expected as urothelial damage occurs with calculus. Dark urine, low specific gravity, and low pH can represent dehydration due to pain common in patients with extensive calculi.

Parameter	Result	Reference range	Interpretation
Appearance	Dark, clear	Clear	Normal/possible dehydration
Specific gravity	1.03	1.005-1.025	Elevated consistent with dehydration
pH	5.5	5.5-8.0	Slightly acidic
Blood	3+	Negative	Haematuria
Protein	1+	Negative	Consistent with urothelial damage
WBC	1+	Negative	Trace amounts
Nitrite	Negative	Negative	Normal
Glucose	Negative	Negative	Normal
Bilirubin	Negative	Negative	Normal
Ketone	1+	Negative	Normal/ dehydration

Diagnostic cystoscopy revealed complete obstruction of the penile and bulbar urethra by multiple stones. The calculi were extracted using forceps, and residual fragments were fragmented and removed using laser lithotripsy. The remaining stones in the bulbar urethra were pushed into the bladder for removal. A large calcified bladder mass was visualised and completely excised via transurethral resection. Histopathological analysis demonstrated WHO 1975 Grade 3/WHO 2004 high-grade papillary urothelial carcinoma, stage pT2, with muscularis infiltration but no lymphovascular invasion.

The patient received intravesical mitomycin C 24 hours following resection prior to histology as immediate postoperative chemotherapy to reduce the risk of tumour cell implantation and early recurrence. The postoperative course was uneventful, and the patient was discharged on day three. He had a follow-up MRI of the pelvis which showed no evidence of locally advanced tumour or pelvic lymphadenopathy and no evidence of benign prostatic hyperplasia. A follow-up computed tomography of the urinary tract showed no evidence of stones in the upper tract, no masses or stones in the ureters, and no evidence of hydronephrosis with complete resection of bladder mass. He remains under observation to undergo regular urine cytology, cystoscopies, and imaging, and to be advised on lifestyle changes. Further workup for deciding on radiotherapy or bladder resection is in progress.

## Discussion

PUC is a common histological subtype of bladder cancer and usually presents with painless haematuria [1,2]. However, atypical presentations may occur when coexisting pathology obscures the diagnosis, as in this case, where extensive urethral and bladder calculi masked an underlying muscle-invasive tumour.

Although the association between bladder calculi and high-grade papillary urothelial carcinoma is uncommon, chronic irritation, infection, and inflammation are recognised contributors to malignant transformation of the urothelium [3,4]. The patient’s laboratory results likely reflected local inflammation secondary to mechanical obstruction rather than systemic infection, as he remained afebrile with stable renal function.

Transurethral resection of the bladder tumour remains the gold standard for diagnosis and initial management [2,5]. Early postoperative instillation of intravesical mitomycin C within 24 hours of resection significantly reduces early tumour recurrence by destroying residual malignant cells and preventing reimplantation [6,7]. Guidelines also recommend intravesical therapy for high-risk non-muscle-invasive disease to decrease recurrence and progression [5,8]. In this case, timely administration of mitomycin C was well tolerated, although histology showed muscle invasion.

Given the tumour’s high-grade, muscle-invasive nature, close surveillance is essential [9]. Follow-up typically includes cystoscopy, urine cytology, and imaging every three months during the first year [10]. Lifestyle modifications, such as adequate hydration, limiting protein and sodium intake [11], regular exercise [12], smoking cessation [13] and moderation of alcohol intake, are essential. At six weeks post-resection, the patient remains asymptomatic with no evidence of recurrence.

This case highlights the diagnostic challenge of distinguishing between benign and malignant pathology in patients with chronic stone disease. Clinicians should maintain a high index of suspicion for malignancy in patients with atypical or recurrent calculi, particularly when imaging reveals complex or calcified intravesical lesions [3,4].

## Conclusions

PUC can rarely present as extensive urethral and bladder calculi, leading to diagnostic delays and underestimation of disease severity. This case highlights the need for thorough endoscopic evaluation and histopathologic assessment in patients with chronic or atypical urolithiasis. Early resection is essential to minimise recurrence risk and improve long-term outcomes.

This report has limitations in that urine cytology was not obtained at the time of presentation, which may have provided additional diagnostic information to complement the histopathological findings. In addition, the follow-up duration is relatively short, restricting our ability to comment on long-term oncologic outcomes such as recurrence or progression. Despite these limitations, this case highlights an exceptionally rare presentation of muscle-invasive papillary urothelial carcinoma associated with extensive urethral and bladder calculi and underscores the importance of maintaining diagnostic vigilance in patients with atypical or recurrent stone disease.
